# Development of a Broadly Accessible Venezuelan Equine Encephalitis Virus Replicon Particle Vaccine Platform

**DOI:** 10.1128/JVI.00027-18

**Published:** 2018-05-14

**Authors:** Sudhakar Agnihothram, Vineet D. Menachery, Boyd L. Yount, Lisa C. Lindesmith, Trevor Scobey, Alan Whitmore, Alexandra Schäfer, Mark T. Heise, Ralph S. Baric

**Affiliations:** a Department of Epidemiology, University of North Carolina at Chapel Hill, Chapel Hill, North Carolina, USA; b Department of Microbiology and Immunology, University of Texas Medical Branch, Galveston, Texas, USA; c Department of Genetics, University of North Carolina at Chapel Hill, Chapel Hill, North Carolina, USA; d Department of Microbiology and Immunology, University of North Carolina at Chapel Hill, Chapel Hill, North Carolina, USA; University of Pittsburgh School of Medicine

**Keywords:** aged, coronavirus, norovirus, VEE replicon, VRP, heterologous, vaccine

## Abstract

Zoonotic viruses circulate as swarms in animal reservoirs and can emerge into human populations, causing epidemics that adversely affect public health. Portable, safe, and effective vaccine platforms are needed in the context of these outbreak and emergence situations. In this work, we report the generation and characterization of an alphavirus replicon vaccine platform based on a non-select agent, attenuated Venezuelan equine encephalitis (VEE) virus vaccine, strain 3526 (VRP 3526). Using both noroviruses and coronaviruses as model systems, we demonstrate the utility of the VRP 3526 platform in the generation of recombinant proteins, production of virus-like particles, and *in vivo* efficacy as a vaccine against emergent viruses. Importantly, packaging under biosafety level 2 (BSL2) conditions distinguishes VRP 3526 from previously reported alphavirus platforms and makes this approach accessible to the majority of laboratories around the world. In addition, improved outcomes in the vulnerable aged models as well as against heterologous challenge suggest improved efficacy compared to that of previously attenuated VRP approaches. Taking these results together, the VRP 3526 platform represents a safe and highly portable system that can be rapidly deployed under BSL2 conditions for generation of candidate vaccines against emerging microbial pathogens.

**IMPORTANCE** While VEE virus replicon particles provide a robust, established platform for antigen expression and vaccination, its utility has been limited by the requirement for high-containment-level facilities for production and packaging. In this work, we utilize an attenuated vaccine strain capable of use at lower biocontainment level but retaining the capacity of the wild-type replicon particle. Importantly, the new replicon platform provides equal protection for aged mice and following heterologous challenge, which distinguishes it from other attenuated replicon platforms. Together, the new system represents a highly portable, safe system for use in the context of disease emergence.

## INTRODUCTION

Vaccines represent critical preparedness platforms for protecting overall public health. Because many newly discovered viruses originate as zoonotic precursors, the key antigenic determinants essential for protective immunity remain unknown, oftentimes until after an outbreak of disease is detected in human populations. Outbreaks of severe acute respiratory syndrome coronavirus (SARS-CoV) in 2002 to 2003 (1, 2) and Middle East respiratory syndrome coronavirus (MERS-CoV) in 2012 (3, 4) are examples of new human viruses which emerged from zoonotic precursors and produce serious disease, especially in aged populations. The recent discovery of SARS and MERS-like CoVs circulating in bats further indicate the ongoing threat to human populations (5–7). Design of a broadly cross-protective vaccine in such instances is dependent upon not only knowledge of the key antigenic determinants but also the availability of a rapid-response vaccine platform to easily generate, characterize, and successfully apply to vulnerable populations, like the aged (8, 9).

Alphavirus replicon particles (VRPs) based on the Trinidad strain of Venezuelan equine encephalitis (VEE) virus are replication incompetent, induce strong mucosal, cellular, and humoral immune responses and ensure clinical safety (10–12). VRPs consist of the VEE virus replicon RNA encoding the replicase proteins and a 26S promoter from which foreign transgenes are expressed (13). As replicon RNAs lack the structural genes, helper RNAs are provided in *trans* to produce infectious particles that infect a wide variety of target cell lines and host species, allowing for vaccination against the heterologous expressed protein (14–16). The absence of preexisting immunity to VEE virus in human populations also provides added utility for the vaccine platform's use in the general population (13, 15, 17, 18).

VRPs based on wild-type VEE virus 3000 as well as attenuated VEE virus 3014 structural protein coats induce robust immunity against a variety of antigens and have proven to be successful vaccine platforms (15, 17, 18). However, concerns have hampered VEE virus 3000-based vaccine applications because of the potential for replication-competent wild-type VEE virus infectious particles. Produced by recombination or from copackaging of helper and replicon RNAs, the VEE virus 3000 VRPs retain some risk of wild-type VEE virus infection or disease (19). For increased safety, VRP vaccines for human use in preclinical and clinical trials have often employed the attenuated VEE virus coat 3014 (14, 18). VEE virus 3014 coat protein differs from the wild type by three amino acids which attenuate VEE virus *in vivo*, possibly by enhancing binding to heparin sulfate, thereby leading to accelerated clearance of virus from the blood (20). VRPs based on 3014 coat have been shown to be successful in inducing immune response and have demonstrated protection in both small- and large-animal models of viral disease (8, 17, 18). Furthermore, the attenuated platform provides ease of purification for manufacturing processes. Despite this attenuation, the VRP 3014 platform still retains some risk for VEE virus-mediated disease and as such must be packaged and safety tested under biosafety level 3 (BSL3) conditions.

SARS-CoV, MERS-CoV, seasonal and pandemic strains of influenza virus, and other pathogens cause disproportionate disease outcomes in the elderly. Mortality rates are found to be ∼50% in SARS-CoV- and MERS-CoV-infected aged individuals (11, 17, 21). Thus, vaccination of aged individuals is imperative to reduce the overall morbidity and mortality of these pathogens. Previous studies from our laboratory have demonstrated that VRP 3014 provides only partial protection from heterologous virus challenge in young animals (11, 17). However, it failed to protect aged animals from lethal homologous and heterologous virus challenge, using SARS-CoV and influenza virus as models (11, 17). This incomplete protection and failure to protect immunosenescent animals was directly correlated with an inability to induce protective antibody responses *in vivo* (8, 11). In parallel studies, VRP 3000-based vaccines also provided incomplete protection but significantly reduced lethal disease and serious clinical disease in aged animals (17). Thus, the attenuation of VRP 3014 produced a platform less effective in aged animals and is therefore less attractive for use in humans, given the expanding aged population over the next 2 decades.

In order to improve the replicon vaccine platform, we generated a new VRP system utilizing the VEE virus 3526 backbone. VEE virus 3526, a live attenuated vaccine strain, is highly immunogenic in primates and horses and is safe when administered by intraperitoneal infection (22). VEE virus 3526 contains a cleavage site deletion in E3 and a second-site resuscitating mutation in E1, leaving the wild-type E1, E2, and capsid sequences intact (23); these mutations are associated with the attenuated phenotype observed for this strain (24). In this work, we describe the generation and characterization of the VRP platform based on VEE virus vaccine strain 3526. We confirm VRP expression of transgenes and the ability to produce virus-like particles for noroviruses. In addition, we tested VRP 3526 expressing the SARS-CoV spike glycoprotein, finding similar antigen expression of spike expressed from the VRP 3000 system. Similarly, the VRP 3526-based SARS spike protected both young and aged mice from lethal disease and correlated with no weight loss, reduced viral replication, and generation of neutralizing antibodies against homologous and heterologous strains. Importantly, VRP 3526 spike was equivalent to VRP 3000 spike and superior to VRP 3014 spike in protecting aged mice from lethal acute respiratory disease outcomes. Taken together, our data demonstrate the highly portable and safe VRP 3526 platform confers a robust expression/vaccine system that induces robust immune responses that protect both young and aged animal models from lethal challenge.

## RESULTS

### Construction and characterization of a VRP 3526 vector platform.

**FIG 1:**
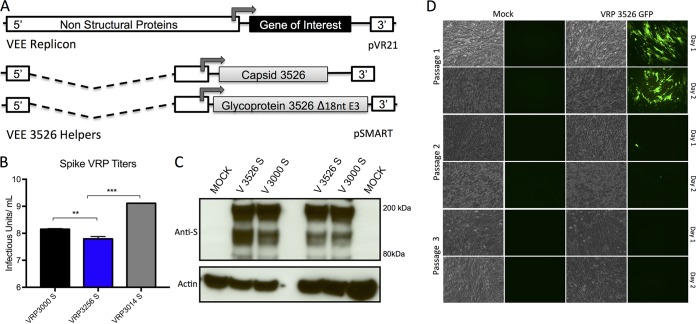
Characterization of VRP 3526 platform. (A) Schematic of VRP 3526 platform showing replicon gene with reporter gene in pVR21 plasmid. Arrows indicate the start of 26S subgenomic promoter. (B) Titers of S protein vaccines from V3000, V3526, and V3014 coats determined on BHK cells by an IFA assay. **, *P* < 0.01; ***, *P* < 0.001 (Student's *t* test). (C) Western blot from independent experiments showing expression of SARS-CoV Spike protein from V3526S and V3000S vaccines in Vero cells. The lower panel shows actin.

While alphavirus replicon particles based on wild-type VRP 3000 or the attenuated VRP 3014 surface coats are highly successful vaccine platforms (15, 17, 24, 25), a myriad of concerns have limited their use, including select agent/BSL3 containment, reversion, and inefficacy of the vaccine platform in vulnerable populations. To overcome these issues, we developed a platform that utilizes the VEE virus 3526 vaccine backbone, which combines the deletion of the entire furin cleavage site between E3 and E2 with a secondary site resuscitating mutation in E1 (23, 26, 27); this attenuated virus packages fused E3/E2 protein (PE2), has been used in humans as a vaccine candidate, and is a BSL2 pathogen (28, 29). Based on this attenuated VEE virus strain, we generated a new VRP vector platform (Fig. 1A). The system uses the sequence of the wild-type VEE 3000 virus, including the 5′ and the 3′ untranslated regions (UTR) (13). The structural glycoproteins (GPs) for VRP 3526 have a deletion in the furin cleavage site in E3 (Δ56RKRR59) that attenuates pathogenesis in cell culture and mice; a second mutation in the E1 glycoprotein restores efficient growth in BHK cells and prevents reversion to virulence through other secondary mutations (23). The glycoprotein genes as well as viral capsid gene were inserted into separate helper constructs between the 26S subgenomic promoter and the beginning of the 3′ UTR. All three plasmids were linearized, and RNA transcripts were electroporated into BHK cells. After 24 to 48 h, cell culture supernatants were collected, the VRPs were purified, and titers were determined on BHK cells, as described before (17). Consistent with a mild packaging defect, VRP 3256 titers ranged between 2.0 × 10^7^ and 1.0 × 10^8^ IU/ml, which was about 1 to 1.5 logs less efficient than the VRP 3000 and VRP 3014 coats (Fig. 1B). Importantly, no evidence for viable recombination and electroporation has been observed with the VRP 3526 system. To date, 47 replicons have been generated and were negative for cytopathic effect (CPE) after passages 1 and 2 (data not shown). To further examine the possibility for replicative virus, two stocks of VRP 3526 replicons expressing green fluorescent protein (GFP) were used to infect Vero cells, subsequently passaged, and examined for CPE and GFP expression (Fig. 1C). Following initial passage, Vero cells showed robust expression of GFP and minimal CPE at days 1 and 2. After the second passage, only a few GFP-positive cells and no CPE were observed over the first 2 days; the final passage showed no evidence of GFP or CPE, suggesting that no viable virus was present in any of the independent passages. Together, the results argue that recombination resulting in viable attenuated VEE 3526 virus is unlikely.

**FIG 2:**
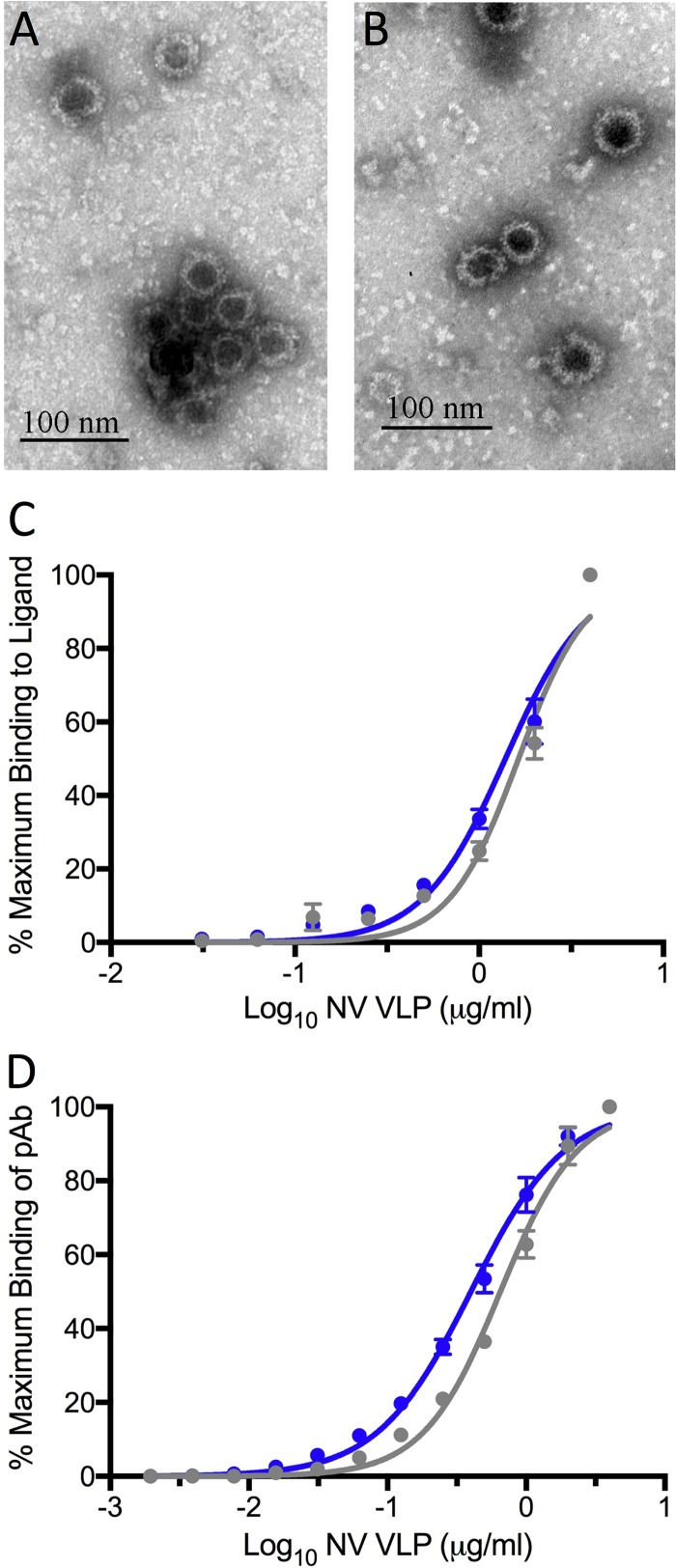
Morphology, ligand binding, and antigenicity of Norwalk virus VLPs produced from V3014 or V3526 VRPs. (A and B) Electron micrograph of Norwalk virus VLPs produced from V3014 (A) or V3526 (B) VRPs. (C) Carbohydrate ligand binding of Norwalk virus VLPs from V3526 (blue) and V3014 (gray). (D) Mouse anti-Norwalk virus polyclonal serum binding to Norwalk virus VLPs. Plotted markers represent the means and standard deviations.

We next characterized antigen expression from the VRP 3526 platform using two model antigens: the SARS CoV spike glycoprotein and norovirus (NoV) VP2 capsid protein. Using the VRP 3000 platform as a control, we compared the expression of SARS-CoV S glycoprotein from the VRP isolated under BSL2 (V3526S) to that isolated under BSL3 (V3000S). Following Vero cell infection, two independent experiments demonstrated that V3526S produced amounts of SARS-CoV S glycoprotein similar to those of V3000S (Fig. 1D). Similarly, NoV VLPs derived from BSL2 V3526-VP1 or BSL3 V3014-VP1 were indistinguishable from each other in morphology, forming ∼40-nm particles with characteristic cup-shaped surfaces (Fig. 2A and B). In addition, particle microstructure integrity was comparable between the 3525 and 3014 VRP platforms based on ligand binding (50% effective concentration [EC_50_] of 1.6 μg/ml for V3014-NoV VLP and EC_50_ of 1.4 μg/ml for V3526-NoV VLP) (Fig. 2C) and mouse polyclonal sera (EC_50_ of 0.65 μg/ml for V3014-NoV VLP and EC_50_ of 0.40 μg/ml for V3014-NoV VLP) (Fig. 2D). Taken together, these data indicate that the VRP 3526 platform yields antigen expression similar to that of previous VRP platforms with the added utility of use at a lower biosafety level.

### Efficacy of VRP 3526 vaccine in young mice.

**FIG 3:**
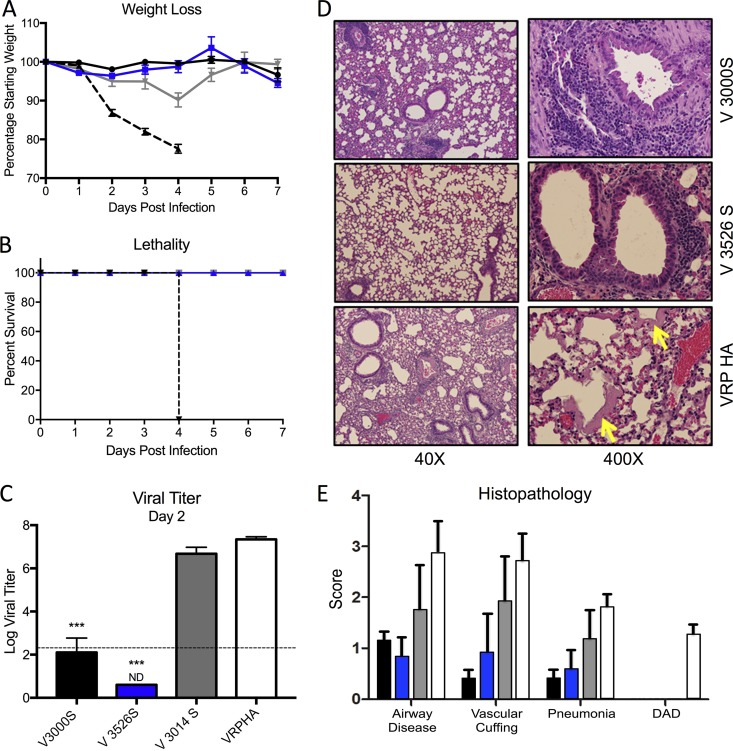
V3526S protects young mice from lethal SARS-CoV disease induced by homologous challenge. (A and B) Percent weight loss (A) and survival curve (B) of young mice immunized with S protein-based vaccines from V3000S (black; *n* = 11), V3526S (blue; *n* = 11), V3014S (gray; *n* = 11), or control (VRP-HA; dashed line; *n* = 11) challenged with 10^5^ PFU of SARS-CoV MA15. (C) Lung virus titers on 2 days postinfection determined by plaque assay on Vero cells (*n* = 5 for VRP, *n* = 3 for control). The dashed line represents the limit of detection for plaque assay. Error bars, SEM; ND, none detected; ***, *P* < 0.001 (Student's *t* test). (D) Representative H&E-stained lung sections harvested 4 days postinfection from the indicated vaccine groups showing lungs, alveoli, and airway vasculature at lower (40×) and higher (400×) magnification. Note the presence of hyaline membranes (yellow arrow) in animals vaccinated with VRP-HA, indicating end-stage lung disease, which is absent from V3000S and V3526S vaccine groups. (E) Scoring of clinical disease in H&E-stained lung sections harvested 4 days postinfection with V3000S (black), V3526S (blue), V3014S (gray), or control (VRP-HA; white) for airway disease, vascular cuffing, pneumonia, and diffuse alveolar damage (DAD).

We next analyzed VRP 3526 spike (V3526S) vaccine performance in young mice in parallel with VRP 3000 spike (V3000S) and VRP 3014 spike (V3014S). Groups of young mice were vaccinated and boosted 3 weeks later with 1 × 10^5^ IU of each VRP vaccine and a control VRP 3000 HA (VRP-HA) encoding influenza H1N1 hemagglutinin. Blood was collected at 21 days postprime, and 3 weeks postboost animals were challenged with a lethal dose of mouse-adapted SARS-CoV (10^5^ PFU rMA15) (17, 21, 30). Animals vaccinated with V3000S and V3526S were completely protected from SARS-CoV-induced disease, as measured by weight loss, morbidity, and death (Fig. 3A). In contrast, V3014S-vaccinated animals lost about 10% of their body weight by day 3 postinfection and then recovered. VRP-HA-vaccinated animals lost 20% of their body weight by day 4 postinfection and experienced severe symptoms of acute respiratory distress syndrome (ARDS) disease (Fig. 3A and B). Analysis of virus titers at 2 days postinfection revealed that animals vaccinated with V3000S and V3526S had minimal viral replication, whereas V3014S-vaccinated animals were not protected from viral replication, similar to VRP-HA-vaccinated animals (Fig. 3C). While all three VRP coats protected young adult mice from end-stage lung disease, mice vaccinated with VRP-HA revealed clear progression to diffuse alveolar damage and ARDS, as indicated by the presence of hyaline membranes (Fig. 3D, lower right, yellow arrow). In contrast, V3526S-vaccinated mice had minimal airway disease and an absence of major lung pathology based on histology scoring (Fig. 3E), similar to the V3000S vaccines, and a modest decrease relative to V3014S. Together, the results demonstrated that the V3526S platform produced protection similar to that of wild-type V3000S and V3014S following homologous challenge.

### VRP 3526 vaccine offers protection from heterologous challenge.

**FIG 4:**
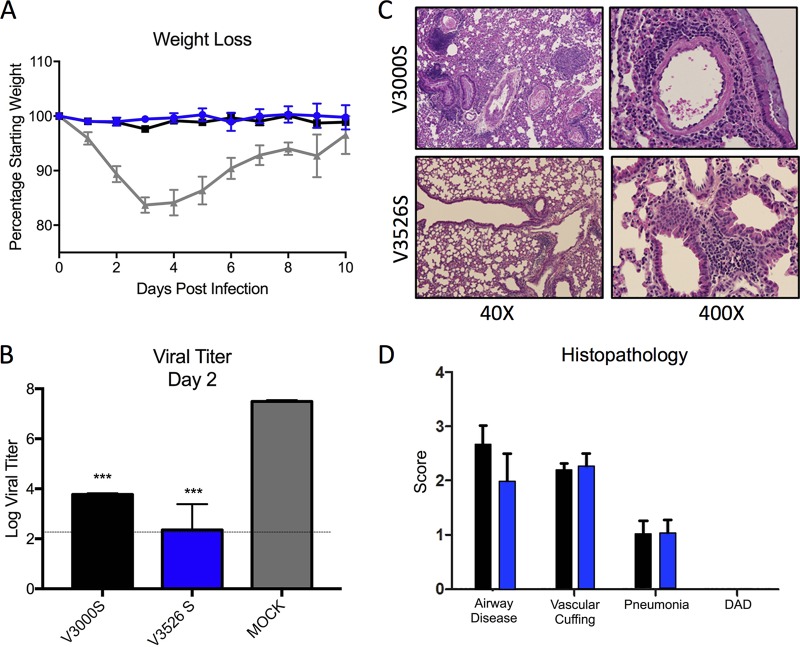
V3526S protects young mice from SARS-CoV disease induced by heterologous challenge. (A) Percent weight loss of young mice immunized with S protein-based vaccines from V3000S (black; *n* = 11), V3526S (blue; *n* = 15), or mock (gray; *n* = 7) immunization and challenge with 10^5^ PFU of rMA15-GD03. (B) Lung virus titers at 2 days postinfection determined by plaque assay on Vero cells with V3000S (black; *n* = 3), V3526S (blue; *n* = 4), or mock (gray; *n* = 3) immunization. Error bars indicate standard errors of the means (SEM). ***, *P* < 0.001 (Student's *t* test). (C) Representative H&E-stained lung sections from V3000S- or V3526S-vaccinated mice harvested 4 days postinfection showing lungs, alveoli, and airway vasculature at lower (40×) and higher (400×) magnification. (D) Scoring of clinical disease in H&E-stained lung sections harvested 4 days postinfection from the indicated V3000S (black)- or V3526S (blue)-vaccinated mice for airway disease, vascular cuffing, pneumonia, and diffuse alveolar damage (DAD). Error bars indicate standard deviations (SD).

We also examined the efficacy of V3526S in protecting young mice from heterologous SARS-CoV challenge. Previous work had shown only modest efficacy of the V3014 platform relative to V3000 following heterologous CoV challenge. In this study, V3000S- and V3526S-vaccinated animals were challenged with a SARS-CoV chimera encoding a spike protein from a heterologous strain, GD03 (17). This mouse-adapted strain had previously been shown to cause 15% weight loss in young animals and significant airway disease (17). Animals immunized with V3000S and V3526S were protected from GD03-MA15-induced weight loss (Fig. 4A), whereas mock-vaccinated animals lost ∼15% of their body weights. Analysis of lung viral titers 2 days postinfection revealed that viral replication was reduced 10,000-fold compared to that of mock-vaccinated animals (Fig. 4B). Notably, histopathology scoring indicated that neither of the platforms abrogated early-stage disease symptoms induced by a heterologous challenge virus (Fig. 4C and D, GD03-MA15); both show improvement relative to previous studies with infected, unvaccinated mice (17). While paling in comparison to protection from homologous challenge (Fig. 3E, MA15), the 3526 platform exceeded the limited efficacy of V3014 in heterologous challenge (17) and represents an improved, safer approach for vaccine generation and development.

### VRP 3526 vaccine platform protects aged mice from lethal SARS-CoV challenge.

**FIG 5:**
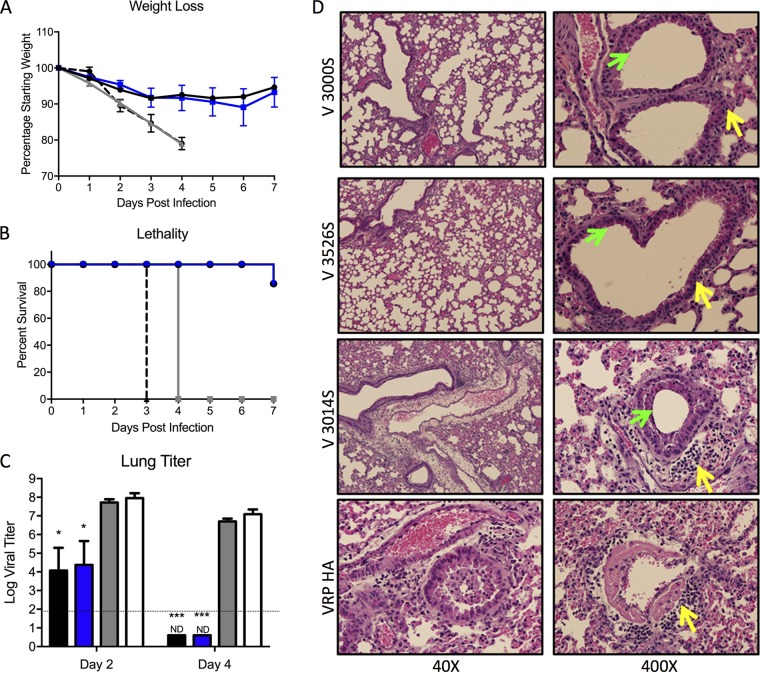
V3526S protects aged mice from lethal SARS-CoV disease. (A to C) Percent weight loss (A) and survival curve (B) of aged mice immunized with S protein-based vaccines from V3000S (black; *n* = 10), V3526S (blue; *n* = 12), V3014S (gray; *n* = 6), or control (VRP HA; dashed; *n* = 7) challenge with 10^5^ PFU of SARS-CoV MA15. (C) Lung virus titers at 2 days postinfection and 4 days postinfection, determined by plaque assay on Vero cells. V3000S (black; *n* = 6 [day 2], 3 [day 4]), V3526S (blue; *n* = 5 [day 2], 3 [day 4]), V3014S (gray, *n* = 3), and control (VRP HA; white; *n* = 4 [day 2], 3 [day 4]) are shown. Error bars indicate SEM. *, *P* < 0.05; ***, *P* < 0.001 (Student's *t* test). (D) Representative H&E-stained lung sections harvested 4 days postinfection from the indicated vaccine groups showing lungs, alveoli, and airway vasculature at lower (40×) and higher (400×) magnification. Note clean airways in V3526S and V3000S groups, as indicated by green arrows, and denuded airways in V3014S (green arrow). Note the massive inflammatory infiltrates (yellow arrows) in V3014S and VRP-HA groups, which are reduced in V3526S and V3000S groups.

We have previously demonstrated that VRP 3000-based, but not VRP 3014-based, vaccines are effective in protecting aged mice from lethal SARS-CoV challenge but not disease (17). We next evaluated the performance of the VRP 3526 vaccine platform in protecting aged mice from lethal SARS-CoV challenge. Groups of 1-year-old animals were vaccinated and boosted with 1.0 × 10^5^ IU of each VRP-S vaccine or control (VRP-HA). Serum was collected 3 weeks after prime and boost to analyze antibody responses, and animals were challenged with 1.0 × 10^5^ PFU of mouse-adapted SARS-CoV (17). Both V3526S and V3000S protected aged mice from severe SARS-CoV-induced disease, as evidenced by minimal weight loss and lethality (Fig. 5A and B). In contrast, animals that received V3014S lost greater than 20% of their body weight 4 days postinfection, died, and/or were euthanized (Fig. 5A and B). Lung virus titers 2 days postinfection (Fig. 5C) revealed virus replication in V3526S-immunized animals was similar to that of V3000S-immunized animals but significantly reduced compared to that of V3014S- or VRP-HA-vaccinated animals. Virus replication was cleared by day 4 postinfection in V3000S- and V3526S-vaccinated aged animals, whereas the viral titers remained high in animals vaccinated with V3014S or VRP-HA. In both V3000S- and V3526S-vaccinated animals, lung pathology showed little evidence of denuded airway cells or airway debris (Fig. 5D, 400×, airways marked by green arrows) with minimal inflammatory cell infiltrates (marked by yellow arrows). In contrast, animals that received V3014S or VRP-HA showed severe airway inflammation with a denuding bronchiolitis (marked by green arrows) with accumulation of apoptotic debris and severe peribronchovascular cuffing characterized by high numbers of lymphocytes, neutrophils, and macrophages (marked by yellow arrows). Similar to the young mouse data, these results indicate superior protection provided by the V3526 S platform in aged mice relative to that of V3014S.

### Protection in young mice and aged mice correlates with antibody response.

**FIG 6:**
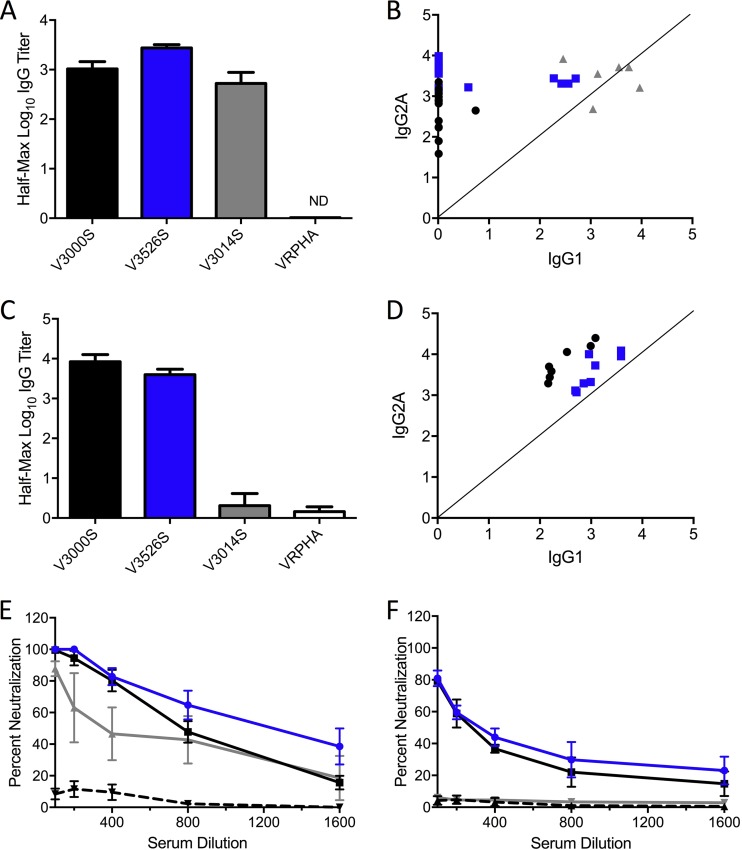
V3526S induces high antibody titers that neutralize SARS-CoV. (A) ELISA results showing IgG titers to S protein, elicited in young mice following vaccination with V3000S (black), V3526S (blue), V3014S (gray), or control VRP-HA (white). (B) Th1/Th2 skewing as measured by IgG_2A_/IgG1 ratio in young mice. (C) ELISA results for aged mice following vaccination by indicated vaccine groups. (D) Th1/Th2 skew in aged mice as measured by IgG_2A_/IgG1 ratios. (E and F) Neutralization potential (SARS-CoV) of antibodies elicited by indicated vaccine group, i.e., V3000S (black), V3526S (blue), V3014S (gray), or control VRP-HA (dashed), in young mice (E) and aged mice (F), as measured by PRNT_50_ assay. ***, *P* < 0.001 (Student's *t* test) relative to V3000S and V3526S.

We next analyzed the serum from young and aged immunized animals. Enzyme-linked immunosorbent assay (ELISA) indicated that the SARS-CoV vaccines elicited high IgG response to S glycoprotein in young animals, regardless of coat (Fig. 6A). Importantly, the V3526S platform elicited serology responses that were equivalent to those measured with V3000S. V3014S vaccine had slightly but significantly lower antibody titers than V3526S and may contribute to reduced protection of young animals from SARS-CoV-induced disease (Fig. 3A). Examining IgG subtypes, the VRP 3014 platform resulted in a mix of both IgG_2A_, associated with a T_h1_ response, and IgG1, associated with a T_h2_ response (Fig. 6B). In contrast, VRP 3000 produced a strong IgG_2A_ response with minimal S-specific IgG1 observed. For VRP 3526, a mixture of IgG_2A_ versus IgG1 responses was observed. For several mice, IgG_2A_ was dominant (5/9); in contrast, several others produced both IgG1 and IgG_2A_. Notably, V3526S vaccination produced a lower magnitude of IgG1 than V3014S and higher IgG_2A_ yields than V3000S. Examining aged mice, only V3526S and V3000S elicited high antibody titers (Fig. 6C), whereas V3014S and VRP-HA proved to be inefficient in eliciting a detectable serologic response. Notably, while both VRP 3526 and 3000 platforms make IgG_2A_, the response is skewed, with more production of IgG1 than is seen with the young mice (Fig. 6D). Together, the V3526S-vaccinated sera from young mice and aged mice indicate augmented specific IgG production and skewing toward IgG_2A_ compared to the V3014 platform.

Extending our analysis, we examined neutralizing antibody responses after vaccination, using serum and 50% plaque reduction neutralization assay (PRNT_50_) against SARS-CoV (17, 31, 32). PRNT_50_ analysis indicated that V3526S elicited high neutralizing antibodies in young mice (PRNT_50_ values of ∼1:988) (Fig. 6E), replicating a phenotype similar to that seen with V3000S (PRNT_50_ values of ∼1:679) (Fig. 6E). PRNT_50_ titers were found to be 3-fold lower in V3014S-vaccinated animals (∼1:286), which likely contributes to partial protection seen after homologous or heterologous challenge. In addition, V3526S was still efficient in eliciting neutralizing antibodies in aged mice (PRNT_50_ titers of ∼1:239) (Fig. 6F), similar to what was observed in V3000S-vaccinated animals (PRNT_50_ titers of ∼1:216). In contrast, there was little if any neutralizing response in animals vaccinated with V3014S. Together, the results clearly indicate that a neutralizing immune response developed after vaccination with the V3256S vaccine and demonstrate it is the preferred platform for future vaccination strategies.

## DISCUSSION

Alphavirus replicon particles based on wild-type VRP 3000 or the attenuated VRP 3014 surface coats are highly successful vaccine platforms (15, 17, 24, 25). However, several disadvantages exist, including (i) select agent restrictions, (ii) packaging and safety testing under BSL3 conditions, (iii) potential generation of wild-type VEE virus due to RNA recombination, (iv) potential generation of replication competent viruses, and (v) failure of VRP 3014 vaccines to protect aged populations against homologous and heterologous virus challenge (17). Strategies to circumvent this problem have included the use of multiple helper RNAs, alphavirus replicon-based adjuvants, or RNA- and DNA-based replicon launch platforms in the absence of structural proteins (13, 33–35). In the manuscript, we have developed and characterized the VRP 3526 platform as a highly portable and safe vector platform for expression of novel genes with features similar to those of the earlier VRP 3000 and VRP 3014 iterations. Importantly, the VRP 3526 platform provided robust protection to vulnerable populations and in the context of heterologous challenge. Coupled with increased safety aspects, the VRP 3526 system represents a significant advance with the potential to be deployed against a myriad of emerging microbial pathogens.

Generated at a lower biosafety level, the attenuated VRP 3526 significantly expands the number of groups capable of using this platform. Both select agent rules and concerns over reversion to wild-type virus had rendered VRP 3000 and VRP 3014 systems inaccessible to the majority of research groups. By incorporating mutations within the backbone and helper constructs, the VRP 3526 platform overcomes these safety concerns and allows manipulation at BSL2. In addition, the VRP 3526 platform had only modest attenuation in terms of titers relative to controls, likely due to incorporated mutations in E1 glycoprotein (13, 23). Importantly, the VRP 3526 platform was able to produce high concentrations of both NoV VP1 and SARS-CoV S glycoprotein. For SARS-CoV, V3526S produced levels of spike antigen similar to those of V3000S. For NoV, the Norwalk VLPs produced within the VRP 3526 system were morphologically and antigenically similar to VLPs expressed from VRP 3014, providing a platform for the manufacture of VLP vaccine candidates. With only minor differences noted in EC_50_s of polyclonal antibodies, the new platform improves on an invaluable tool needed to study genetic determinants of antigenic drift and escape and to design a successful norovirus vaccine. Together, the results suggest that the VRP 3526 system can replicate VRP 3000 and 3014 as an amplification platform for microbial antigens.

The ability to provide *in vivo* protection against lethal challenge from human pathogens is an appealing feature of the VRP platform and allows for rapid preclinical testing of vaccines. *In vivo* studies in young mice immunized with V3526S demonstrated clear protection from SARS-CoV challenge. In contrast, the V3014 coat provided suboptimal protection in terms of weight loss, viral replication, and damage to the lung in young mice. Similarly, the VRP 3526 platform provided protection equivalent to that of VRP 3000 in the context of heterologous challenge. While the 3014 platform had previously been shown to fail (17), challenge with GD03-MA15 resulted in reduced weight loss, viral replication, and disease in mice vaccinated with V3526S. Notably, protection correlated with generation of high-titer, antigen-specific antibodies, and the VRP 3526 platform produced antibody results similar to those for V3000. Although not directly tested, these data suggest that overall B- and T-cell responses are improved in VRP 3526 vaccination compared to those in VRP 3014 vaccination. Together, the results indicated that the safer VRP 3526 platform outperformed the VRP 3014 platform, providing protection following both homologous and heterologous CoV challenges.

Since emerging viruses cause disproportionate disease burdens in the elderly, successful vaccination of aged individuals with VRP 3526 represents a key public health intervention strategy. V3526S was efficient in protecting aged mice from lethal SARS-CoV challenge and was indistinguishable from V3000S. Neutralizing antibodies played a major role in protection, as evidenced by neutralizing titers of V3526S similar to titers measured for V3000S vaccine (17) and 3- to 4-fold higher than thresholds needed for protective immunity (21). This was also evidenced by the clearance of virus from lungs at late times postinfection, which in part could be due to T-cell-mediated responses which have been shown to play a protective role (36, 37). Consistent with our previous study (11, 17), V3014S vaccines failed to elicit protection in aged individuals, performing similar to the control, VRP-HA. Importantly, V3526S induced high antibody titers and neutralization in the aged, which were absent from V3014S and suggest diminished overall adaptive immune responses. While these responses skewed toward IgG1, the virus-specific antibody response corresponded with similar results offered by V3000S and provided a mechanism for protection in aged mice. Together, the data clearly demonstrate VRP 3526 is an improved, attenuated VRP vaccine platform for aged mice.

Since the emergence of SARS-CoV at the beginning of the century, zoonotic diseases have posed a significant threat to global public health. Highly portable and safe vaccine platforms are key in rapidly responding to these emerging threats. In this study, we describe a significant advance to the VEE virus replicon particle (VRP) systems that makes the platform more accessible. The attenuated VRP 3526 system provides an opportunity to use this resource at a lower biosafety level, and the safety incorporated within the system does not sacrifice either antigen production or protection under several vaccine conditions. The overall result is a platform that can be rapidly applied to new, emerging diseases with efficacy in vulnerable populations and in the context of heterologous challenge.

## MATERIALS AND METHODS

### Ethics statement.

This study was carried out in strict accordance with the recommendations for care and use of animals by the Office of Laboratory Animal Welfare (OLAW), National Institutes of Health. The Institutional Animal Care and Use Committee (IACUC) of The University of North Carolina at Chapel Hill (UNC; permit number A-3410-01) approved the animal study protocol followed in the manuscript. Animals were anesthetized with ketamine and xylazine (per IACUC, UNC, guidelines) for infection and were euthanized if the body weight dropped below 80% of starting weight or clinical symptoms warranted it per IACUC, UNC, guidelines. UNC is registered with the Centers for Disease Control and Prevention to work with select agents, including SARS-CoV.

### Virus and cells.

The titers of recombinant a SARS-CoV Urbani strain, icSARS (GenBank accession no. AY278741), a mouse-adapted derivative, rMA15 (21, 30), and a mouse-adapted derivative encoding a heterologous S glycoprotein, rMA15 GD03-S (17), were determined, and they were propagated on Vero E6 cells as previously described (17, 38). rMA15 differs from the epidemic strain SARS-CoV Urbani in 6 amino acids (H133Y nsp5, E269A nsp5, T67A nsp9, A4V nsp13, Y436H spike, and E11K M protein) (GenBank accession no. AY525636). The GD-03 strain of SARS-CoV was identified from sporadic human cases during the second wave of the SARS epidemic in 2004 and is distinct from the 2003 Urbani-derived epidemic isolates (11, 17). rMA15 GD03-S was derived from an MA15 molecular clone by replacing the MA15 Urbani spike with the GD03-S glycoprotein encoding the Y436H mouse-adapted S mutation (17). Vero E6 cells were grown in minimal essential medium (MEM; Invitrogen, Carlsbad, CA) supplemented with 10% FetalClone II (HyClone, South Logan, UT) and gentamicin-kanamycin (UNC Tissue Culture Facility). All SARS-CoV work was performed in a class II biological safety cabinet in a certified biosafety level 3 laboratory containing redundant exhaust fans. Personnel wore Tyvek suits, gloves, and shoe covers and used portable air breathing apparatus (PAPR) as described previously (39). All animal work was performed in SealSafe HEPA-filtered mouse caging as specified by the manufacturer (Techniplast) using IACUC-approved protocols and procedures (40).

### Packaging and production of VRPs expressing SARS-CoV glycoproteins from different coats.

VRPs expressing the SARS-CoV spike glycoprotein from VEE virus 3000, 3014, or 3526 coat were constructed as previously described and assembled under identical conditions (11, 17). For packaging VRP 3526, genes encoding the capsid and envelope proteins were synthesized from BioBasic, Inc., and were inserted into pSMART vectors (Lucigen Inc.). VRP construct expressing SARS-CoV S glycoprotein was generated using overlap PCR by fusing an amplicon containing the S gene in frame with an amplicon containing sequences from the VEE virus replicon. The primers for the VEE virus replicon and the primers used for generating the S gene amplicon were described previously (41). VRP-HA utilizes the V3000 coat and was generated as previously described (17). Ligated DNA was digested with ApaI and PacI and inserted into the pVR21 plasmid. VRPs were packaged using helper RNAs encoding structural proteins as described before (41).

### Generation of polyclonal mouse antisera, neutralization assays, and Western blot analysis.

Five-week-old mice were primed and boosted with 10^5^ infectious units (IU) of the VRPs. Following vaccination, mouse polyclonal sera were generated from BALB/c mice as described previously (17). Neutralization assays using mouse antisera involving SARS-CoV were performed as described previously (17). For Western blotting, lysates from cells infected with VRP 3526 S or VRP 3000 S were prepared as described before in detail (42), and these blots were probed using the indicated mouse polyclonal sera.

### Mice immunizations and challenge with lethal SARS-CoV challenge.

Ten and 50 2-week-old female (BALB/c) mice were primed at 5 weeks or 12 months of age, respectively, with 1 × 10^5^ IU of each respective VRP or mock vaccinated with phosphate-buffered saline (PBS) as a control. Titers of VRP were determined in Vero cells using antisera against the replicase proteins. Mice were monitored for 7 days after prime to ensure that unexpected complications (e.g., virus-induced hind leg paralysis or encephalitis) that could arise as a result of the generation of authentic VEE virus by recombinatory processes *in vivo* did not occur. Serum was collected by the tail nick method 21 days after prime (8, 17). The animals were boosted again with 1 × 10^5^ IU of VRPs and monitored daily as described previously. Twenty-one days postboost, serum was again collected by tail nick for analysis. All of the animal immunizations were performed under BSL2 conditions. Animals were transferred and allowed to acclimatize for at least 1 week in the BSL3 laboratory to avoid any stress prior to virus challenge. Animals were anesthetized with ketamine-xylazine mixture and intranasally infected with 10^5^ PFU of rMA15 virus or rMA15 GD03 S in a 50-μl volume. Animals were weighed daily, and clinical symptoms of disease were monitored regularly. Animals that lost >20% of their body weight were euthanized according to the IACUC guidelines. Lung tissues were harvested 2 and 4 days postinfection to analyze viral titers, histopathology, and viral and host transcripts that change as a result of vaccination/challenge.

### Determination of lung titers.

Briefly, portions of the lung were weighed, placed in 0.5 ml Dulbecco's PBS (DPBS), and frozen at −80°C until analyzed. Lungs were then homogenized in a Magna Lyzer (Roche) and clarified by centrifugation (12,000 rpm, 1 min in a microcentrifuge), and virus titers within lung supernatants were assessed via plaque assay in Vero E6 cells as described previously (8). Virus pathology was assessed as previously described by our group (43).

### Pathological evaluations.

Lungs harvested 2 and 4 days postinfection were fixed in 4% formaldehyde (formalin) for 7 days to ensure virus inactivation. The formalin was replaced using fresh formalin before the tissues were transferred out of BSL3 for processing under BSL2 conditions. The lung tissues were then embedded in paraffin, and cut tissue sections were stained with hematoxylin and eosin (H&E) to identify any immune infiltrates in the lung. Pathology from H&E-stained sections were blind scored for clinical disease features, including airway disease, vascular cuffing, pneumonia, and diffuse alveolar damage, on a scale of 0 to 3 (1, mild; 3, severe).

### Norwalk virus-like-particles.

To determine whether the VRP 3526 platform could produce sufficient protein for the production of VLP-based vaccines, the Norwalk virus ORF2 gene was inserted directly into the VEE virus replicon vector for the production of VRPs as previously described (17). VLPs were expressed in VRP-infected BHK cells and purified by velocity sedimentation in sucrose, followed by simultaneous concentration and dialysis into PBS using 100-kDa-cutoff centrifugal filter units (Millipore) (44).

### EIAs.

Mouse anti-Norwalk virus VLP polyclonal serum reactivity to Norwalk virus VLPs was determined by enzyme immunoassay (EIA) (45). Briefly, plates were coated with decreasing concentrations of VLP in PBS before the addition of 0.2% serum. Primary antibody incubation was followed by anti-mouse IgG-horseradish peroxidase (HRP) (GE Healthcare) and color development with one-step ultra TMB ELISA HRP substrate solution (Thermo-Fisher). Each step was followed by washing with PBS–0.05% Tween 20, and all antibodies were diluted in 5% dry milk in PBS–0.05% Tween 20. The percent maximum binding was defined as the binding level (optical density at 450 nm) at a given VLP concentration divided by the binding level of 1 μg/ml VLP multiplied by 100. Data were fit with sigmoidal dose-response curves in Prism 6 (GraphPad), and EC_50_s were calculated. EC_50_s between VLPs generated from different VRP coats were compared using a Student's *t* test. A difference was considered significant if the *P* value was <0.05.

### VLP-carbohydrate ligand-binding assays.

Pig gastric mucin type III (PGM; Sigma Chemicals) was solvated in PBS at 5 mg/ml, coated onto EIA plates at 10 μg/ml in PBS for 4 h, and blocked overnight at 4°C in 5% dry milk in PBS–0.05% Tween 20 before decreasing concentrations of Norwalk virus VLPs were added for 1 h. Bound VLPs were detected by a mouse anti-Norwalk VLP monoclonal antibody at 2 μg/ml followed by anti-mouse IgG-HRP (GE Healthcare), and color was developed with one-step ultra TMB ELISA HRP substrate solution (Thermo-Fisher). All incubations were done at room temperature. Each step was followed by washing with PBS–0.05% Tween 20, and all reagents were diluted in 5% dry milk in PBS–0.05% Tween 20. Data were analyzed as described above for EIAs.

### Statistical approaches.

Statistical analysis was performed using Prism (GraphPad, San Diego, CA) software. Nonparametric Mann-Whitney or Student's *t* tests were performed to generate *P* values as noted in the text.
